# Early Detection of Cartilage Degeneration: A Comparison of Histology, Fiber Bragg Grating-Based Micro-Indentation, and Atomic Force Microscopy-Based Nano-Indentation

**DOI:** 10.3390/ijms21197384

**Published:** 2020-10-06

**Authors:** Bastian Hartmann, Gabriele Marchi, Paolo Alberton, Zsuzsanna Farkas, Attila Aszodi, Johannes Roths, Hauke Clausen-Schaumann

**Affiliations:** 1 Center for Applied Tissue Engineering and Regenerative Medicine–CANTER, Munich University of Applied Sciences, 80335 Munich, Germany; bastian.hartmann@hm.edu (B.H.); Attila.Aszodi@med.uni-muenchen.de (A.A.); 2 Laboratory of Experimental Surgery and Regenerative Medicine (ExperiMed), Clinic for General, Trauma and Reconstructive Surgery, University of Munich, 82152 Planegg, Germany; Paolo.Alberton@med.uni-muenchen.de (P.A.); Zsuzsanna.farkas@med.uni-muenchen.de (Z.F.); 3 Center for Nanoscience (CeNS), University of Munich, 80331 Munich, Germany; 4 Photonics Laboratory, Department of Applied Sciences and Mechatronics, Munich University of Applied Sciences, 80335 Munich, Germany; Gabriele.marchi@web.de (G.M.); roths@hm.edu (J.R.)

**Keywords:** FBG sensor, FBGindentation-type atomic force microscopy, FBGearly detection of osteoarthritis, FBGoptical biosensor, FBGbiomechanics of cartilage

## Abstract

We have determined the sensitivity and detection limit of a new fiber Bragg grating (FBG)-based optoelectronic micro-indenter for biomechanical testing of cartilage and compared the results to indentation-type atomic force microscopy (IT-AFM) and histological staining. As test samples, we used bovine articular cartilage, which was enzymatically degraded ex vivo for five minutes using different concentrations of collagenase (5, 50, 100 and 500 µg/mL) to mimic moderate extracellular matrix deterioration seen in early-stage osteoarthritis (OA). Picrosirius Red staining and polarization microscopy demonstrated gradual, concentration-dependent disorganization of the collagen fibrillar network in the superficial zone of the explants. Osteoarthritis Research Society International (OARSI) grading of histopathological changes did not discriminate between undigested and enzymatically degraded explants. IT-AFM was the most sensitive method for detecting minute changes in cartilage biomechanics induced by the lowest collagenase concentration, however, it did not distinguish different levels of cartilage degeneration for collagenase concentrations higher than 5 µg/mL. The FBG micro-indenter provided a better and more precise assessment of the level of cartilage degeneration than the OARSI histological grading system but it was less sensitive at detecting mechanical changes than IT-AFM. The FBG-sensor allowed us to observe differences in cartilage biomechanics for collagenase concentrations of 100 and 500 µg/mL. Our results confirm that the FBG sensor is capable of detecting small changes in articular cartilage stiffness, which may be associated with initial cartilage degeneration caused by early OA.

## 1. Introduction

Osteoarthritis (OA) is one of the most frequent joint diseases worldwide. At an early stage, cartilage degradation starts at the molecular level, primarily affecting the structural and mechanical properties of the articular cartilage surface [1,2]. Cartilage deterioration is accompanied by degradation of its extracellular matrix (ECM) constituents essentially involving proteoglycans and the collagen fibrils, which gradually impairs the mechanical function of the tissue. In vitro, changes in molecular composition and integrity of the articular cartilage ECM can be detected, e.g., by histochemistry and immunohistochemistry, while tissue structural and mechanical properties can be assessed by atomic force microscopy (AFM) [3,4]. However, in vivo detection of structural and biomechanical changes in early OA remains challenging. Especially during arthroscopy, the surgeon is currently dependent on the localization of the degenerated tissue by optical examination and by palpating the cartilage surface by hand or by using a hook [5,6]. This presupposes a great experience of the surgeon and shows high variations resulting from the subjective judgment of the examiner. This underscores the need for measurement devices that can support the medical evaluation of the cartilage condition [7]. To address this problem, different test-devices have been developed [8,9,10,11,12]. The applied probes have diameters of about 1–3 mm and lengths of several centimeters, which makes endoscopic integration difficult.

To provide a tool for identifying degenerated tissue more reliably and at an earlier stage, we recently presented a fiber Bragg grating (FBG)-based micro-indenter which has the potential to be used as a minimally invasive endoscopic device during arthroscopy [13,14]. We have previously demonstrated that the sensor is capable of determining the viscoelastic properties of cartilage and to discriminate healthy and heavily degenerated cartilage [15]. In the present study, we determined its sensitivity and investigated if the sensor is able to detect minor cartilage degenerations associated with early OA and thus provides clinically relevant results. For this assessment, we compared the results of the FBG sensor gained on mildly degenerated bovine cartilage with indentation-type atomic force microscopy (IT-AFM) and histological Safranin O/Fast Green staining of the same samples. Histology is an established and commonly used clinical tool for the detection and classification of OA in human and animal models. Histopathology, applying the most frequently used Mankin’s system [16] or the Osteoarthritis Research Society International (OARSI) system [17], grades structural and biochemical changes associated with the progression of articular cartilage degradation, adds an important in vitro extension of radiological and arthroscopic evaluation of OA but does not provide biomechanical information of the diseased tissue. IT-AFM, on the other hand, is currently one of the most sensitive tools for measuring mechanical changes of ex vivo cartilage samples and is capable of detecting the stiffness reduction associated with the onset of OA in both humans and experimental animal models [4,18,19]. Previous studies have also investigated the capability of directly using an AFM integrated into a device for the arthroscopy of knee cartilage [20,21]. However, this approach using an AFM-like setup during arthroscopy has some disadvantages such as high temperature drift and mechanical instability when used inside the joint of a patient. Furthermore, the observable area is very limited, which can be an issue when it comes to the task of mapping over large areas like a whole condyle. The FBG sensor can provide a more robust method for accessing the biomechanical properties of knee cartilage of patients and could also allow for the comparably fast mapping of larger cartilage surface areas [22]. Because the FBG sensor is based on glass fibers, already used, e.g., for visualization purposes during surgeries, it has the potential to be integrated into existing arthroscopic devices.

In order to achieve controlled degradation of ECM constituents in cartilage plugs mimicking human OA, several in vitro enzymatic digestion protocols have been developed. Collagenase or elastase treatments induce the degradation of cartilage-specific collagens, which in turn also facilitates some loss of sulfated proteoglycans, e.g., aggrecan, from the disintegrated fibrillar network [23,24]. In contrast, direct degradation of proteoglycans can be achieved by chondroitinase ABC, trypsin or cathepsin D digestions [24,25]. To investigate the consequence of in vitro ECM breakdown on cartilage biomechanics, Stolz et al. enzymatically depleted collagens or proteoglycans in porcine articular cartilage, and the cartilage stiffness was investigated by nano-scaled IT-AFM [24]. The study demonstrated that collagen depletion by prolonged elastase treatment (1–2 days) lowers cartilage stiffness, while digestion of the proteoglycans by cathepsin D results in stiffening compared to control, undigested cartilage. Similarly, we have previously reported that FBG-based micro-indentation resolved stiffness reduction of severely degraded bovine articular cartilage, in which proteoglycan depletion was achieved by 12 h of trypsin digestion [15].

In the present study, we exposed bovine articular cartilage explants to short, time-fixed digestion of collagenase at various concentrations to mimic the very early stages of OA. We report that nano-scaled IT-AFM was superior to detect cartilage softening induced with the lowest collagenase concentration but failed to clearly discriminate between mechanical changes induced by higher collagenase concentrations. In contrast, FBG-based micro-indentation was less sensitive than IT-AFM but was still able to resolve stiffness reduction in the higher collagenase concentration range. Thus, we conclude that an FBG sensor has the capability to detect mild degradation of the articular cartilage and, once integrated into an arthroscopic device, could assist mechanical evaluation of early osteoarthritic cartilage in a clinical set-up.

## 2. Results

**Figure 1:**
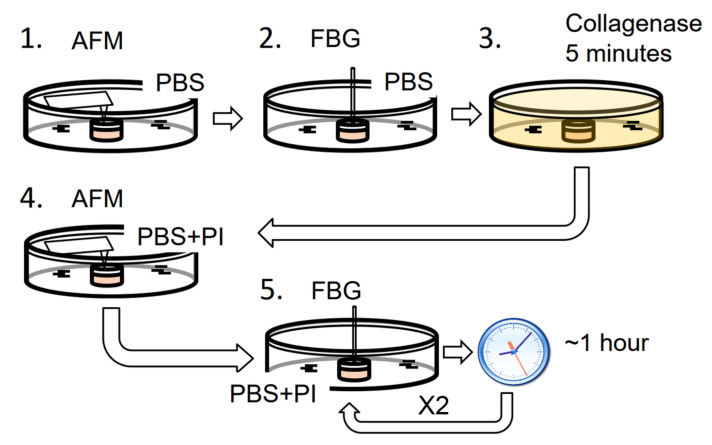
Measurement procedure: 1. The native thawed sample is measured by indentation-type atomic force microscopy (IT-AFM); 2. The sample is then subject to a stress relaxation measurement by the fiber Bragg grating (FBG) micro-indenter; 3. The sample is digested five minutes in a collagenase solution; 4. After rinsing, the digested sample is immersed in phosphate-buffered saline (PBS) with protease inhibitor (PI) and measured again by IT-AFM approximately at the same locations as the previous measurement; 5. The digested sample is again measured by FBG micro-indenter at the same position as the previous measurement three times with an interval of one hour between the measurements.

To compare the sensitivity of the fiber-based micro-indenter with the sensitivity of IT-AFM, the measurement procedure depicted in Figure 1 was used. In brief, to determine the stiffness values of the native cartilage, the untreated samples were first investigated by IT-AFM, followed by the FBG micro-indenter. Next, the cartilage was degenerated with collagenase and investigated again, first by IT-AFM and then by FBG indentation (three measurements at 1-h intervals, see also Section 4.3.).

### 2.1. FBG Results

**Figure 2:**
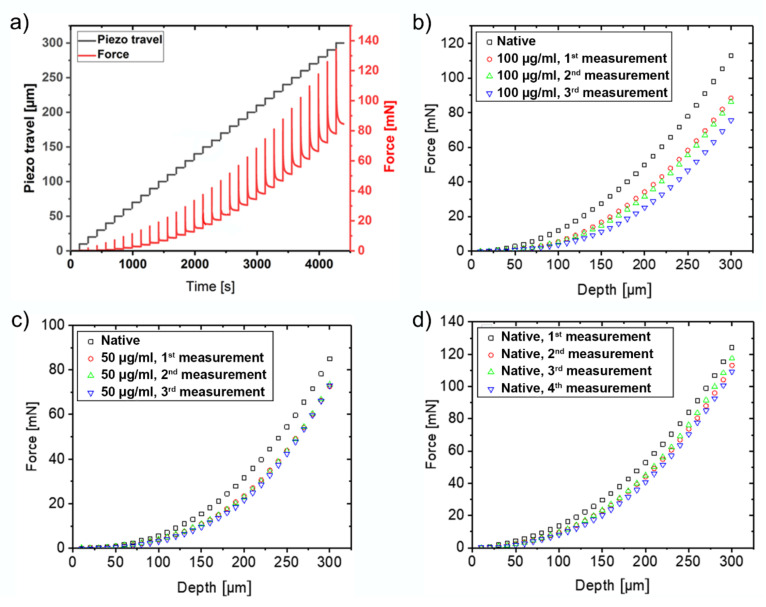
(**a**) Piezo travel (black line) and stress-relaxation curve (red line) as a function of time for a native cartilage sample. It is particularly evident that the initial non-linear part of the indentation curve (toe region) becomes linear with depth. The next graphs show the indentation curves for (**b**) a sample treated with 100 µg/mL concentration of collagenase, (**c**) a sample treated with 50 µg/mL collagenase and (**d**) an untreated sample.

Figure 2a depicts a typical indentation curve obtained with the FBG sensor on native (untreated) femoral condyle bovine cartilage. The black curve shows the step-wise piezo movement vs. time and the red curve shows the corresponding stress-relaxation curve. The force vs. indentation curves illustrated in Figure 2b–d were derived from the stress-relaxation curves, by taking the force at the end of each indentation step, after the compressed cartilage had relaxed to its new equilibrium position, as described in detail in Marchi et al. (2018) [26]. The black squared data points in Figure 2b,c represent the indentation on native untreated cartilage (step 2 in Figure 2). The red, green and blue data points represent the three consecutive measurements on collagenase treated samples. The measurements on collagenase treated samples were repeated in one-hour intervals, while the sample remained in the measurement setup without changing its position. Both for the treatment with 100 µg/mL (Figure 2b) and with 50 µg/mL collagenase solution (Figure 2c), a clear difference can be seen between the curves obtained on native and on collagenase treated cartilage, while the differences between the three subsequent measurements on the treated samples are much smaller. As controls, we performed four consecutive measurements at one-hour intervals on another piece of untreated cartilage (Figure 2d). Even here, a slight shift between the different measurements can be observed, which is particularly pronounced between the first and second measurement. This shift may be caused by the solvation of small ECM molecules or fragments, by ion exchange between the PBS buffer and the cartilage sample, or by the measurement procedure itself. The shift between the first and second measurement is comparable to the stiffness reduction observed between untreated samples and samples treated with 50 µg/mL collagenase and thus currently limits the sensitivity of our FBG system to the treatment with approximately 50 µg/mL collagenase.

**Figure 3:**
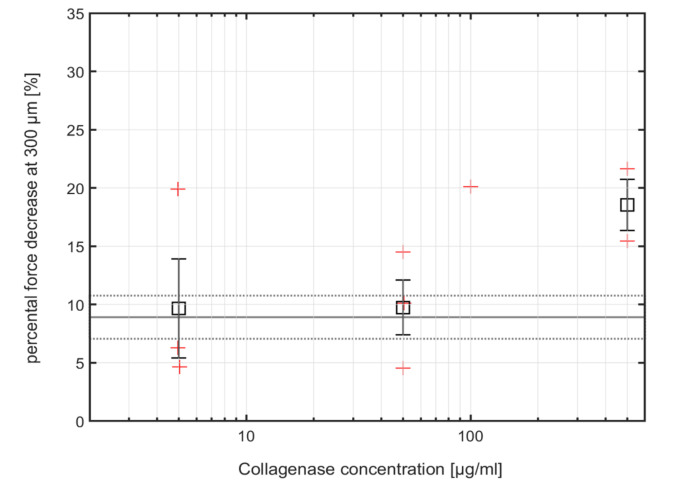
Percentage force differences at different collagenase concentrations in µg/mL and means of the results measured with the FBG micro-indenter. The grey solid line indicates the mean for the data measured on the native cartilage (concentration 0 µg/mL) which is not displayed, and the grey dotted lines indicate the standard error of this mean. The red crosses indicate individual measurements and the black squares are the corresponding mean values with error bars indicating the standard error of the means. At the concentration of 100 µg/mL, only one data set (FGB and IT-AFM) is available, therefore no mean value and standard error were calculated for this concentration. The *p*-values of a one-way unbalanced ANOVA test against native sample variation for 5, 50, and 500 µg/mL are 0.87, 0.79, and 0.06, respectively.

In general, at indentation depths between 100 and 300 µm, all indentation curves measured on a sample before the digestion showed higher force values and a faster ascending trend than the curves measured on the same sample after digestion. Due to the variability of the cartilage between individual animals as well as along the condyle surface of the same animal, the absolute force value at 300 µm indentation was quite variable from sample to sample. Therefore, we adopted the relative difference of the force at 300 µm indentation between the native and digested condition of each sample, i.e., the force difference divided by the force value measured at 300 µm on the native sample, as an indicator of cartilage degeneration. Figure 3 shows the relative differences of the forces (in percent) for the individual cartilage samples (red crosses) between the native state compared to the first measurement after digestion, as well as the mean values (black squares) and standard errors (black bars) for the different collagenase concentrations. The grey lines in Figure 3 indicate the relative differences obtained between repeated measurements on untreated samples (8.8% ± 1.9%, mean (solid) and standard error (dotted)) and thus represents the current sensitivity limit of our method. At a collagenase concentration of 5 and 50 µg/mL, the mean of the relative differences obtained on treated and untreated samples is only 9.67% ± 4.26% and 9.74% ± 2.35%, and thus below this sensitivity limit. This is also reflected by *p*-values 0.87, and 0.79 of a one-way unbalanced ANOVA test against native sample variation for 5, and 50 µg/mL, respectively. At a collagenase concentration of 500 µg/mL, however, the relative change of 18.54% ± 2.19% is well above the sensitivity limit of our sensor, indicating that the FBG microindenter is capable of detecting the stiffness decrease induced by 500 µg/mL collagenase treatment. Here, the *p*-value of the ANOVA test reaches 0.06, which is very close to the commonly applied 0.05 threshold of statistical significance, and which could probably be further reduced by recording more data points.

### 2.2. AFM Results

**Figure 4:**
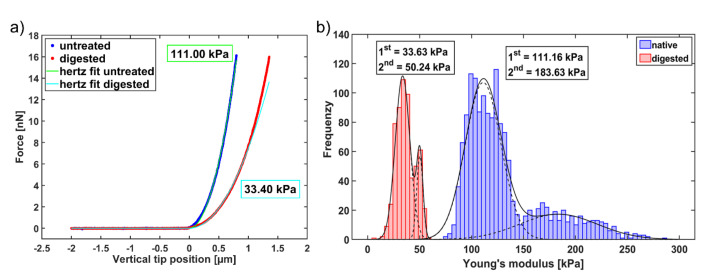
(**a**) Illustration of two representative force-indentation curves obtained by IT-AFM with the corresponding fits of the Hertz model. The blue line represents a force-indentation curve of native bovine cartilage, where the Hertz fit (green line) determines a Young’s modulus of 111.00 kPa. In contrast, the red line represents a force-indentation curve measured on the same cartilage, but after a 5 min digestion with 100 µg/mL collagenase. The Hertz fit (cyan line) determines a reduced Young’s modulus of 33.40 kPa. (**b**) Young’s modulus distributions of a native cartilage sample (blue distribution) and the distribution obtained from the same sample after a collagenase treatment with 100 µg/mL (red distribution). The bimodal fits to the histograms (solid black lines) reveal a softening of the tissue due to the collagenase digestion of about 70% for the 1st peak (from 111.16 kPa to 33.63 kPa) and about 73% for the 2nd peak. The dashed lines in the histograms represent the two individual Gauss distributions of the corresponding bimodal distribution.

**Figure 5:**
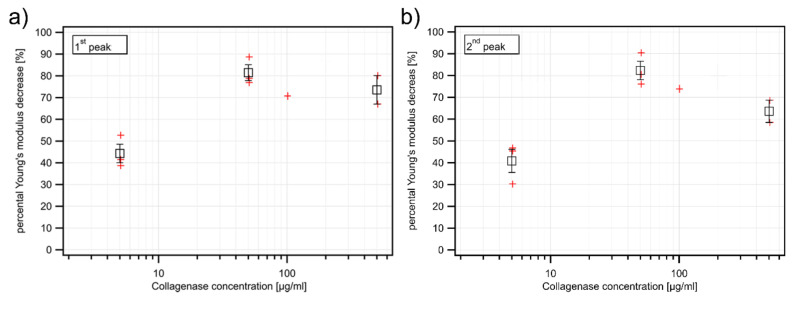
(**a**) Percentage decrease of Young’s modulus at different collagenase concentrations in µg/mL determined with IT-AFM and the means of the results of the 1st peak and (**b**) of the 2nd peak. The red crosses indicate individual measurements and the black squares are the corresponding mean values with error bars indicating the standard error of the means. At the concentration of 100 µg/mL, only one data set (FGB and IT-AFM) is available, therefore no mean value and standard error were calculated for this concentration. The *p*-values of one-way unbalanced ANOVA tests against native sample variation for 5, 50, and 500 µg/mL are 0.005, 0.0002, and 0.0033 for the first peak (a) and 0.0111, 0.0003, and 0.004 for the second peak (b).

Representative AFM force-indentation curves on untreated (blue curve) and degenerated cartilage (red curve) are shown in Figure 4a. From these curves, the Young’s modulus was extracted by fitting the Hertz model for a pyramidal indenter. For the two exemplary curves shown in Figure 4a, treatment with 100 µg/mL collagenase reduces the Young’s modulus of the cartilage from 111.00 kPa to 33.40 kPa. The Young’s moduli of all force curves obtained on this sample before and after collagenase treatment are displayed in the histogram shown in Figure 4b, where the blue bars represent Young’s modulus values obtained on the untreated cartilage and the red bars represent values after the collagenase treatment. To quantify the effect of the collagenase treatment, a bimodal Gaussian distribution was fitted to the blue and red histogram as described in [27] and the positions of the two peaks (1st and 2nd peak), which have been attributed to the proteoglycan and the collagen components of the cartilage, were extracted. Subsequently, the relative decrease of the Young’s modulus between the native and collagenase treated cartilage was calculated for each peak and plotted against the collagenase concentration (Figure 5a, first peak and 5b, second peak). For the example given in Figure 4b, the relative decrease was about 70% for the 1st peak (from 111.16 kPa to 33.63 kPa) and about 73% for the 2nd peak (from 183.63 kPa to 50.24 kPa). This example also shows that the two peaks of the characteristic bimodal distribution found on native cartilage [27] tend to merge after collagenase treatment, which in some cases results in a unimodal distribution.

The results of the AFM experiments, which are summarized in Figure 5a for the first peak and 5b for the second peak, demonstrate that the AFM is capable of detecting the changes in cartilage elasticity induced by the treatment with 5 µg/mL collagenase, which results in an ~40% decrease of the Young’s modulus. At higher collagenase concentrations, the AFM always detects an ~80% decrease of the Young’s modulus. Thus, although the AFM is a very sensitive tool for detecting subtle changes in cartilage biomechanics, it fails to detect differences in more severely degraded samples. This is most likely due to the small indentation depth of only 1 µm which is probed by the AFM, compared to the 300 µm probed by the FBG sensor. It should be noted that the relative differences observed with the AFM during repeated measurements on identical untreated samples are 10.8% ± 4.2% (data not shown), and that the sensitivity limit of the AFM was thus not attained in our experiments.

### 2.3. Histology

**Figure 6:**
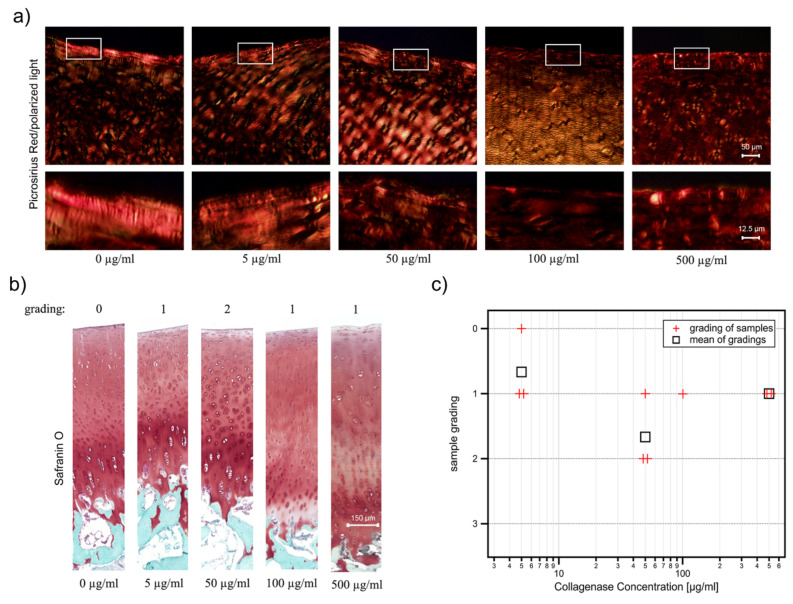
Summary of the histological findings of the measured bovine cartilage samples after the treatment with different concentrations of collagenase. In (**a**) representative images of Picrosirius Red staining of undigested and collagenase digested samples taken with polarized light. Rectangles indicate the magnified superficial zone of the articular cartilage. The collagenase concentrations are given in µg/mL. In (**b**) representative images of the Safranin O/Fast Green stained sections of the average condition at different collagenase concentrations. In (**c**) the grades of all samples are summarized together with the mean values (black squares) per concentration, except for the concentration of 100 µg/mL, where the result of only one sample is available.

The histological stainings of the cartilage samples are shown in Figure 6. Topo-optical reactions using Picrosirius Red for visualizing oriented collagen fibrils under polarized light show that the birefringence, as a sign of intact collagen network, is reduced in the superficial zone of collagenase digested samples compared to control (Figure 6a). We observed a gradual decrease in birefringence from 5 µg/mL to 100 µg/mL collagenase concentrations, while no obvious difference in signal intensities was found between samples treated with 100 µg/mL and 500 µg/mL collagenase. This result confirms that our enzymatic digestion protocol degraded the superficial collagen network in a concentration-dependent manner.

Since the loss of an intact collagen network could affect the retention of other molecules in the articular cartilage ECM [24], we also investigated proteoglycan depletion. The microscope images of the Safranin O-stained sections reveal a partial loss of sulfated proteoglycans in the superficial layer of the degenerated samples (see Figure 6b). As expected after such a mild enzymatic digestion, the surface of the cartilage is still intact and neither cracks nor fibrillation can be seen. Thus, all treated samples correspond to an OARSI grade [17] of 0. Therefore, we evaluated our cartilage samples with a finer scoring system, which takes into account only the depth of proteoglycan loss (see Section 4.4). The scorings of our samples are summarized in Figure 6c. Most samples are graded 1 and the mean values do not show a clear trend from low to high collagenase concentrations. Instead, the mean values vary between a grading of 1 and 2. Thus, the histological staining for proteoglycans is not sensitive enough to differentiate between the degradation levels generated with the collagenase treatment, even when a modified and more sensitive scoring system is applied.

## 3. Discussion

Osteoarthritis is the leading pathological condition of the joint causing disability and pain, which involves articular cartilage degeneration, as well as subchondral bone and synovial membrane changes. The primary functions of articular cartilage, namely resisting compressive, tensional and shear stresses, are gradually compromised during the onset and progression of OA owing to an irreversible breakdown of its extracellular matrix constituents [28]. Proteoglycan and collagen degradation in the articular cartilage is the major biochemical hallmark of injury-induced or age-associated OA, which inevitably affect the morphological and biomechanical properties of the tissue. Thus, modalities allowing structural, biochemical and biomechanical evaluation of the various stages of articular cartilage deterioration have become an essential toolbox for early diagnosis of OA. The morphological alterations underlying OA progression can be precisely monitored through histopathology using evaluation methods such as the OARSI grading and scoring system [17], which, however, require isolation of cartilage biopsies from the patients. Magnetic resonance imaging (MRI) is a powerful, non-destructive tool to detect morphological and ECM compositional differences between healthy and osteoarthritic knee articular cartilage, although MRI is currently not recommended for the diagnosis of early OA in the clinical practice due to the lack of consensus criteria [29].

While obvious morphological changes are not necessarily present in early OA, biomechanical properties of the articular cartilage have been proposed as sensitive indicators of pathophysiological changes occurring already at the initial phase of OA [2,30,31,32]. Especially, nano-indentation tests using high-resolution AFM have demonstrated that mechanical changes precede histological signs of OA [4,19], thus, nano-scaled IT-AFM is an ideal tool to diagnose early OA [33]. As an alternative modality to AFM, we have recently suggested a novel fiber Bragg grating-based micro-indentation system to characterize the biomechanical properties of articular cartilage [13]. We showed that the optical indenter sensor can be used for scanning over comparatively large surface areas [22] and is able to discriminate between healthy and osteoarthritic human articular cartilage [34].

In the present study, we mimicked moderate degradation of the articular cartilage by enzymatic degradation of the collagen network. According to the OARSI histopathological grading system for human knee osteoarthritis, the disease is initiated at the joint surface as manifested by fibrillation or disruption of the superficial zone, which later extends as cracks into the deeper zones when OA is progressing [17]. Usually, the loss of proteoglycans from the surface region is also a sign of initial degeneration which ultimately alters the biomechanics of the articular cartilage. The applied collagenase digestion protocol resulted in mild proteoglycan loss and collagen degradation in the superficial zone, thereby, reflecting the very early changes associated with the initiation of OA. Our comparative measurements show that the FBG micro-indenter is also able to detect the biomechanical changes in bovine articular cartilage caused by limited enzymatic degradation with collagenase. The effect of a five-minute treatment with 500 µg/mL collagenase was still detectable, while treatment with 50 µg/mL collagenase for 5 min was no longer detectable with the FBG sensor. IT-AFM, on the other hand, was able to clearly detect and quantify the Young’s modulus reduction caused by a five-minute treatment with 5 µg/mL collagenase. The FBG micro-indenter is thus less sensitive than IT-AFM, which is the current benchmark for detecting subtle changes in cartilage biomechanics but has the potential to provide a more robust tool regarding environmental influences and for accessing larger areas of the cartilage surface compared to AFM-based arthroscopy techniques [20,21]. With histological staining, however, it was not possible to reliably distinguish between the different grades of degeneration of any of the samples, even with a customized and more sensitive scoring system than the OARSI system [17]. The fact, that the FBG micro-indenter was able to detect all but the mildest degenerations (5 µg/mL and 50 µg/mL), shows its ability to detect relevant alterations of cartilage biomechanics, associated with the onset of OA. This emphasizes that the FBG based sensor has the potential to provide surgeons with additional quantitative data during arthroscopy when integrated into a minimally invasive arthroscopic tool. However, because of the variations of cartilage biomechanics between individual patients and across joints, this will most likely require the 2D scanning of larger areas of the cartilage surface and searching for local stiffness variations caused, e.g., by focal cartilage defects. In a recent study we have demonstrated that by scanning the FBG sensor across a sample surface, it is indeed capable of detecting and quantifying elasticity gradients of the sample [22]. In addition, our results emphasize that the FBG sensor is potentially able to detect early OA even before the surface roughness of the cartilage is increased due to wear-related changes, which is crucial for other AFM-based approaches to detect the onset of OA [35].

Only for IT-AFM at a collagenase concentration of 5 µg/mL was the drop of the Young’s modulus distinguishable from the other concentrations, while all collagenase concentrations above 5 µg/mL led to a decrease of the Young’s modulus of ~80%. The discrimination of the different levels of cartilage degeneration was therefore not possible for higher enzyme concentrations. This is either due to the fact that the maximum level of collagen degeneration attainable by the collagenase used was already achieved within the first 1 µm of the sample, or because the degree of collagen degeneration within this 1 µm was already too severe for the AFM to discriminate any further changes. Thus, the AFM confirmed that digestion of the cartilage had, in fact, occurred at all collagenase concentrations, however, its useful working range is limited to minute cartilage degenerations and small depths, making our FBG sensor potentially a valuable tool to appreciate in vivo early OA-like mechanical changes, as it is capable of indenting several hundred µm into the sample.

## 4. Materials and Methods

### 4.1. Articular Cartilage Sample Preparation

Femoral condyles of an 18-month-old steer were acquired from the local slaughterhouse. Cylindrical osteochondral plugs were harvested from the same condyle with the aid of an 8 mm diameter trephine drill. To keep a record of the anatomical position, a map of the extracted plugs was drawn. Samples were wrapped in phosphate-buffered saline (PBS) pH = 7.4 soaked gauze and stored at −20 °C until used. The sample plugs consisted of the articular cartilage together with the subchondral bone. On the day of measurements, samples were thawed and unwrapped from the gauze. A part of the bone was sawed parallel to the cartilage surface to obtain a sample height of about 3–5 mm. Afterward, the bottom of the sample was glued to a glass Petri dish with Braun Hystoacryl glue (B. Braun Melsungen AG, Melsungen, Germany) and the sample was fully immersed in PBS. After temperature equilibration, the indentation measurements started.

To ensure that the freeze-thaw cycle due to the storage does not alter the biomechanical properties of the cartilage significantly, as has been reported previously [36], we conducted IT-AFM measurements on three different cartilage samples. The first was measured right after the preparation without any freezing, the second and third was measured after a storage time of one and two weeks, respectively. Those measurements showed no significant difference between the freshly prepared and the stored samples (data not shown).

### 4.2. Sample Degeneration

Degeneration of the articular cartilage surface was achieved by collagenase type II digestion (Worthington Biochemical Corporation, Lakewood, NJ, USA, activity: 215 u/mg). The osteochondral plugs were immersed in collagenase dissolved in PBS for five minutes at 37 °C. To obtain different states of degeneration, we applied different collagenase concentrations: 5 µg/mL, 50 µg/mL, 100 µg/mL and 500 µg/mL. After the five minutes, the collagenase solution was discarded, the cartilage was rinsed with PBS and subsequently immersed in PBS supplemented with Complete Protease Cocktail Inhibitor (Roche AG, Basel, Switzerland). The protease inhibitor has been added to block any remaining collagenase activity on the cartilage. Based on preliminary studies using various combinations of collagenase concentration and digestion duration, the time of 5 min was chosen to not severely disrupt the collagen network of the cartilage and to limit ECM degradation to the superficial zone, thereby mimicking the very initial phase of OA.

### 4.3. Measurement Procedure

Samples were firstly measured in their native status immediately after thawing and equilibrating with IT-AFM followed by the FBG sensor. Afterward, samples were degenerated as described in Section 4.2. and measured in three adjacent areas (each 3 × 3 µm^2^ with 25 × 25 force-curves) in the middle of the plug, using indentation-type atomic force microscopy (IT-AFM) to ascertain the effectivity of the degeneration. Immediately after, the sample was measured using the FBG micro-indenter. The location of the indentation was manually aligned to the middle of the plug. To account for time-dependent effects, several FBG measurements were performed at the same position on the degenerated sample at intervals of one hour.

#### 4.3.1. IT-AFM Measurements

Indentation-type atomic force microscopy (IT-AFM) measurements were conducted using a NanoWizard I (JPK Instruments, Berlin, Germany) and data analysis was carried out as described in Alberton et al. (2019) [3]. To position the AFM tip within the region of interest on the sample, the setup was equipped with a long-distance top-view microscope (Nivatar) and a CCD camera (Imaging Source). For vibration insulation, the AFM setup was mounted on a granite slab suspended with rubber bands inside a soundproof box to reduce external noise. The vertical tip velocity during the indentation experiments was 12 µm/s. As a probe, a cantilever with a nominal spring constant of 0.1 N/m and a four-sided pyramidal tip with a tip radius of ~20 nm was used (Cantilever E, MLCT AFM probes, Bruker, Camarillo, CA, USA). The IT-AFM measurements were performed in PBS at pH 7.4 and after the collagenase treatment, protease inhibitor was added to the PBS. For each sample, three force maps were recorded 3 × 3 µm^2^ with 25 × 25 force-curves). The lateral spacing between the three maps was kept minimal (10 – 20 µm) to minimize spatial variations of the sample. For the determination of the Young’s modulus E, the Poisson’s ratio ν was set to 0.5 [24,37,38] and the opening half-angle  α of the tip was 17.5° for our tips. All force-distance curves were analyzed from the contact point to a maximum indentation depth of 1 µm using the modified Hertz-model for a pyramidal indenter [39]:

(1) $$F\left(d\right)=0.7453\frac{E}{\left(1-\nu^{2}\right)}\tan\left(\alpha \right)d^{2}.$$

For the statistical analysis of the obtained Young’s modulus values, the results of the three maps of each sample and degradation stage were merged into a stiffness distribution (histogram). The data analysis was carried out with the JPK data processing software (version 5.0.130) follows the method described by Prein et al. (2016) [27].

#### 4.3.2. FBG Measurements

**Figure 7:**
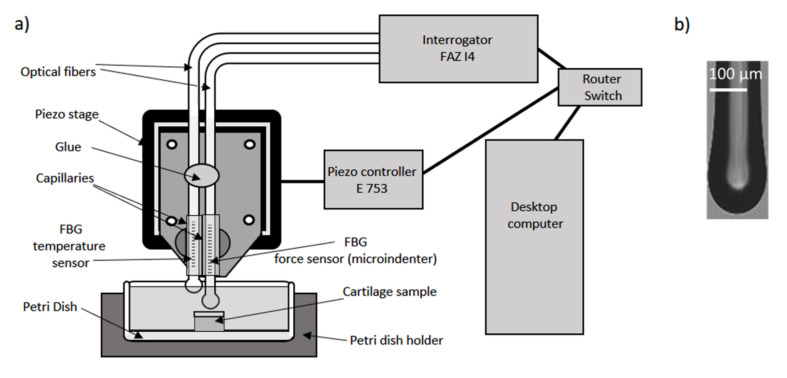
(**a**) Schematic setup used for measuring the stress-relaxation of the cartilage samples. (**b**) Microscope image of the FBG micro-indenter tip.

The measurement principle, the detailed experimental setup, and the general measurement procedure were already presented in Marchi et al. (2017) [13] and in Marchi et al. (2018) [26]. A schematic diagram of the FBG micro-indenter is shown in Figure 7a and the size and shape of the rounded glass fiber used for indentation are depicted in Figure 7b. The micro-indenter comprises a 25 mm long FBG inscribed in a photosensitive optical fiber (GF1B, Nufern, East Granby, USA) of 125 µm diameter. The optical fiber itself was used as the indenter and the tip of the fiber was rounded by arc discharge and had a radius of curvature of 75 µm. The distance between the tip and the FBG was about 2 mm. The FBG sensor was inserted in a glass capillary of 180 µm inner diameter to prevent bending during indentation. The optical fiber and the glass capillary were fixed on an aluminum plate using cement glue. A second FBG sensor, which was fixed adjacent but vertically displaced by ~2 mm to the FBG micro-indenter served as a temperature sensor, in order to compensate thermal drift occurring during the measurement. The Bragg wavelengths of both FBG sensors were determined using a fast interrogator (I4, FAZ technologies, Dublin, Ireland). The aluminum plate with the FBG micro-indenter was fixed on a piezo stage (PI HERA 625.1, Physik Instrumente, Karlsruhe, Germany). The piezo stage was controlled via a Piezo controller (E-753, Physik Instrumente, Karlsruhe, Germany). A Petri dish containing the sample was placed below the micro-indenter. A custom-made notched Petri dish holder was used to ensure that FBG measurements, after AFM measurements and enzymatic degradation, were carried out in the same region on the sample, in order to minimize variation due to spatial variability of the cartilage elasticity. After positioning the sample and approaching the cartilage surface with the micro-indenter, the stress-relaxation measurements were started by stepwise indentation with steps of 10 µm and a dwelling time of 120 s at each step [26]. Thirty steps were performed at each position until the maximum indentation depth of 300 µm was reached. The force *F* exerted by the indenter and recorded by the FBG was calculated according to:

(2) $$F=\frac{\Delta \lambda }{\lambda_{B}}\frac{{\left(d/2\right)}^{2}E}{\left(1-p\right)},$$

where Δλ is the temperature corrected wavelength shift of the indenter FBG, $\lambda_{B}$ is its Bragg wavelength, d is the diameter, p the effective elasto-optic coefficient and E the Young’s modulus of the optical fiber, as described in detail in [13]. Between the three repeated FBG measurements of the digested samples, the samples were left in the FBG setup and only the FBG sensor was vertically retracted using the z-piezo, in order to ensure that the repeated measurements were carried out exactly at the same position.

Typical force data of a stepwise indentation procedure are depicted in Figure 2a. After each step, a partial stress-relaxation is observed. Here, the last value of each relaxation curve, just before the next piezo step (equilibrium force), was picked and plotted against the indentation depth to obtain force-indentation curves, which are shown in Figure 2b–d.

### 4.4. Histological Processing and Scoring System

At the end of each measurement, the plugs were rinsed in PBS and fixed with 95% ethanol and 1% acetic acid in dH_2_O. On the next day, samples were rehydrated in descendent solutions of ethanol/dH_2_O, washed in PBS and decalcified in 10% formic acid/PBS for 3 days at room temperature with mild agitation. Decalcified plugs were thoroughly washed in PBS, and incubated in 20% sucrose/PBS for 24 h at 4 °C. Osteochondral plugs were embedded in Tissue Tek cryomedia (Sakura Finetek, Alphen aan den Rijn, Netherlands), and gradually frozen on a chilled copper plate placed on dry ice. Sagittal sections of 10 µm were cut using a cryotome (HM500 cryostat) and collected on Superfrost Plus glass slides (Thermo Fischer Scientific, Waltham, MA, USA). To visualize collagen, Picrosirius Red staining was performed as follows: rehydrated cryosections were stained for 30 min in 0.1% Sirius red (known as Direct Red 80, 365548, Merck, Darmstadt, Germany) dissolved in saturated picric acid solution. Sections were then dehydrated in three changes of absolute ethanol, cleared in xylene and mounted in Roti-Histokitt mounting medium (cat. num. 6638.1, Carl Roth GmbH, Karlsruhe, Germany). For the examination of proteoglycan depletion, a Safranin O/Fast Green staining was performed, as described in Alberton et al. (2019) [3]. Bright-field and polarized light images were acquired with an AxioVert 40 CFL using a 40× objective and AxioCam 105 color camera (Carl Zeiss, Göttingen, Germany). Cartilage histopathology was initially assessed using the OARSI grading system applying six grades, which reflect the depth of lesions extending from the surface of the articular cartilage. To judge the effect of the moderate collagenase treatments, we generated a customized scoring system, which takes into account only the proteoglycan depletion, as indicated by a decreased Safranin O staining compared to the untreated cartilage. The system comprehends grades 0 to 5, as follows: 0 = no loss of proteoglycans; 1 = loss in the superficial layer; 2 = loss down to the transition layer; 3 = loss down to the deep zone; 4 = loss down to the calcified cartilage; and 5 = loss down to the subchondral bone. The score of each sample was obtained by eye inspection of the bright-field microscope images of the histological sections and subjective evaluation of the chromatic intensity of the Safranin O staining. The staining was assessed by two independent observers blind to the treatment of the samples. 

### 4.5. Statistical Analysis

All values for each collagenase concentration, except 100 µg/mL, represent the mean and corresponding standard error. For the collagenase concentrations 5 and 50 µg/mL, three cartilage samples were measured, and for the concentration of 500 µg/mL two samples were measured. For the concentration of 100 µg/mL, just one measurement result is available. The one-way unbalanced ANOVA tests were carried out using Matlab version R2020a (MathWorks, Natick, MA, USA). 

## 5. Conclusions

In the present study, we have determined the sensitivity and detection limit of a recently developed FBG-based force sensor [13] with respect to its ability to detect mechanical changes caused by cartilage degeneration. We have compared the sensor to IT-AFM and histology, two well-established methods for the detection of biomechanical and structural alteration in cartilage. Although IT-AFM was able to detect cartilage degeneration caused by a 20-fold lower enzyme concentration, than the FBG sensor, the FBG sensor was still able to detect comparatively mild degenerations associated with early OA, which could not be detected by the OARSI histopathological assessment system. The FBG based sensor, therefore, has the potential to provide valuable additional information to surgeons during arthroscopy, when integrated into an arthroscopic device and equipped with an x-, y-scanner enabling the detection of lateral stiffness variations caused by, e.g., focal defect. In addition, for research applications and the characterization of cartilage biomechanics in the laboratory, the FBG sensor provides useful additional information, which is complementary to IT-AFM and has the potential to bridge the gap between AFM based nano-indentation and large scale compression of cartilage, as it is done in conventional mechanical testing [40].

## Abbreviations

AFM	Atomic force microscopy
ECM	Extracellular matrix
FBG	Fiber Bragg grating
IT-AFM	Indentation-type atomic force microscopy
MRI	Magnetic resonance imaging
OA OARSI	Osteoarthritis Osteoarthritis Research Society International
PBS	Phosphate-buffered saline
